# The LVI Component Hypothesis: the lymphatic-to-vascular invasion ratio as an enrichment biomarker for therapy with PD-L1×VEGF-A bispecific antibodies

**DOI:** 10.3389/fimmu.2026.1878891

**Published:** 2026-08-14

**Authors:** Kenneth A. Kern

**Affiliations:** 1 Affiliate Faculty, Skaggs School of Pharmacy and Pharmaceutical Sciences, University of California, San Diego, San Diego, CA, United States; 2 Oncology Breakthrough Advisors LLC, San Diego, CA, United States

**Keywords:** bispecific antibodies, immune checkpoint inhibitors, lymphatic vessels, lymphovascular invasion, predictive biomarker, programmed death-ligand 1, tumor microenvironment, vascular endothelial growth factor A

## Abstract

Lymphovascular invasion (LVI) describes intravasated tumor cells within intratumoral lymphatic or blood vessels. LVI is reported routinely on pathologic examination because it is prognostic for reduced disease-free and overall survival across many solid tumors. In contrast, it is not considered a predictive biomarker because its presence cannot discriminate which patients benefit from a given therapy. To become predictive, a biomarker must reflect the aberrant, specific biology a drug targets. Bispecific antibodies with functional arms against immunologic and vascular targets are a recent class of biologics that simultaneously engage the malignant immunologic and angiogenic components of a tumor. In this setting, deconstructing LVI into its invasive lymphatic and vascular components, and expressing their ratio, is hypothesized to add predictive capability to this histologic finding. LVI-positive tumors are enriched for parameters relevant to both arms of this class of bispecific antibodies. Immunologically, they carry higher tumor mutational burden, more frequent PD-L1 expression, and TGF-β signaling. Angiogenically, blood vessel invasion reflects a VEGF-A-driven, disordered, T-cell-excluding vasculature. The author proposes the LVI Component Hypothesis, which is that tumor cell intravasation into lymphatic versus blood vessels can be expressed as a ratio, the lymphatic-to-vascular invasion ratio (LVR). This ratio may predict benefit from PD-L1×VEGF-A bispecific antibodies. No clinical data currently link the LVR to response, so it is presented as a hypothesis to be tested, first in archived tissue from completed trials of PD-L1×VEGF-A bispecific antibodies and then in prospective randomized trials. If validated, the LVR might help to select those patients most likely to respond, raising the response rate among those treated and possibly sparing those unlikely to benefit.

## Introduction

Lymphovascular invasion (LVI) is the presence of tumor cells within the lumen of a lymphatic or blood vessel on histologic examination, reported as present or absent in tissue biopsies and resected surgical specimens (1, 2). Its presence is an independent indicator of prognosis (reduced disease-free and overall survival) in many solid tumors, including breast, colorectal, and non-small cell lung cancer (3–5). As a prognostic indicator, the presence of LVI portends increased risk, whether or not a patient receives a specific systemic therapy. LVI is not a predictive biomarker, because it cannot as yet identify the patients in whom an anti-tumor agent confers benefit, which requires a treatment-by-biomarker interaction (6–8).

A biomarker becomes predictive when it identifies treatment-relevant biology against which an oncologic therapy acts. For example, EGFR mutation status predicts benefit from osimertinib because the drug’s target is the mutated EGFR protein (9). While LVI identifies tumor cells lying within the intratumoral lumens of lymphatic and blood vessels, in the era preceding immunotherapy, its presence has served only to prognosticate a more aggressive tumor type. However, in the era of immunotherapy, and in particular in the use of certain bispecific antibodies targeting both the immunologic and angiogenic components of tumor biology, LVI might serve as a predictive biomarker. This switch from prognostic to predictive biomarker is not without precedent. HER2 amplification was established as a marker of relapse and reduced survival in 1987 (10), and became predictive only when trastuzumab was tested against it fourteen years later (11).

Bispecific antibodies whose functional arms combine checkpoint blockade with inhibition of vascular endothelial growth factor A (VEGF-A) are in trials across solid tumors. As of late 2025, a landscape analysis identified 56 registered trials of agents directed at both a checkpoint target and VEGF (12). VEGF-A produces disordered tumor vasculature that impairs T-cell infiltration, recruits immunosuppressive myeloid populations, and induces endothelial anergy, each of which blunts checkpoint blockade; VEGF-A inhibition restores vascular normalization and T-cell entry (13–17). Delivering checkpoint inhibition and anti-angiogenic therapy by separate antibodies has already produced clinical benefit in hepatocellular carcinoma, non-small cell lung cancer, renal cell carcinoma, and triple-negative breast cancer (18–22). Placing both activities on a single molecule presumably concentrates them within the tumor microenvironment, where VEGF-A binding by one arm increases local antibody retention and checkpoint occupancy by the other (23). In the phase 3 HARMONi-2 trial, conducted in China, ivonescimab, a PD-1×VEGF-A bispecific antibody, improved progression-free survival compared with pembrolizumab monotherapy at a preplanned interim analysis (median 11.1 vs 5.8 months; stratified HR 0.51, 95% CI 0.38–0.69), demonstrating clinical activity for a PD-1×VEGF-A bispecific antibody (24, 25). The hypothesis developed here concerns the PD-L1×VEGF-A format, exemplified by pumitamig (BNT327/PM8002), now in phase 2 trials in small cell lung cancer and triple-negative breast cancer (26). In the PD-L1 format, the checkpoint arm can bind PD-L1 on lymphatic endothelium itself (27, 28).

Patient selection for this class currently rests on PD-L1 expression alone. HARMONi-2 restricted eligibility to PD-L1-positive tumors without other mutational drivers (25); the registered phase 2 trials of pumitamig apply comparable checkpoint-based criteria (26). PD-L1 immunohistochemistry is an imperfect instrument for this purpose, given its documented variability in staining and scoring across assays and platforms (29). Further compounding the need for a more comprehensive biomarker, no angiogenic or vascular variable has served as an entry criterion in any registered trial of this class, despite the VEGF-directed arm carried by every molecule in it (12, 26).

This article proposes the LVI Component Hypothesis, which posits that the ratio of lymphatic to vascular invasion (LVR) may predict benefit from PD-L1×VEGF-A bispecific antibodies. The LVR would be determined by separating lymphovascular invasion into its lymphatic and vascular components using D2–40 and CD31 immunohistochemistry, with D2–40 serving as the discriminating lymphatic marker, applied to archived tumor tissue according to the criteria described below (32–34). The biological rationale is that tumor cells within lymphatic vessels are in direct contact with PD-L1-expressing lymphatic endothelium (27), whereas tumor cells within blood vessels occupy a VEGF-A-rich perivascular niche (13). LVI positivity is therefore not itself the proposed predictive biomarker, but rather a prerequisite for calculating the LVR, because the ratio cannot be determined in tumors lacking invaded vessels.

## The LVI component hypothesis

**Figure 1:**
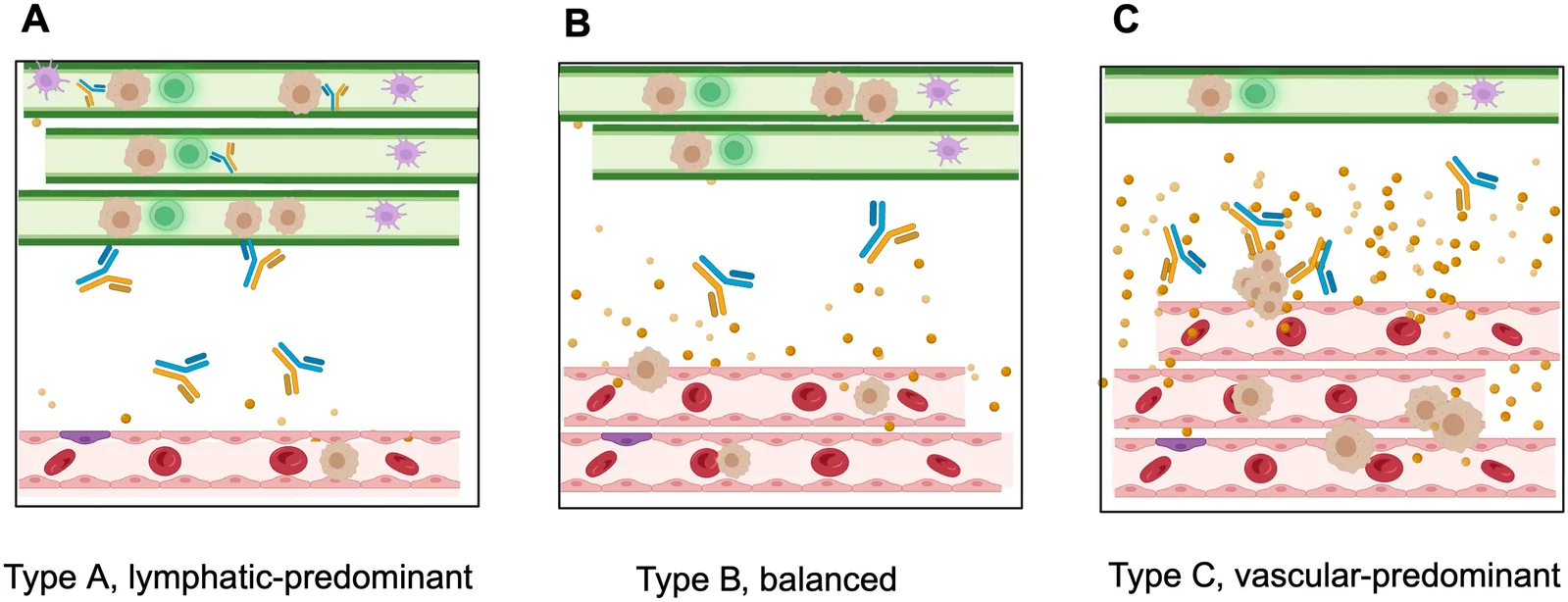
Component-directed lymphovascular invasion and the predicted response to a PD-L1×VEGF-A bispecific antibody. This conceptual schematic illustrates the proposed relationships and does not depict experimentally measured data. Only the PD-L1×VEGF-A format is shown; PD-1-directed agents are not represented. In each panel, D2-40-positive lymphatic vessels with PD-L1-expressing endothelial lining (27) are shown at the top, and D2-40-negative, CD31-positive blood vessels at the bottom. Tan cells, tumor cells; green cells, lymphocytes; purple cells, dendritic cells; red cells, erythrocytes; orange dots, VEGF-A; Y-shaped molecules, PD-L1×VEGF-A bispecific antibodies (blue arm, anti-PD-L1; orange arm, anti-VEGF-A). Tumors are grouped by the lymphatic fraction of invaded vessels, L/(L+V), where L is the number of invaded lymphatic vessels and V is the number of invaded blood vessels. The proposed preliminary categories are Type A, >0.67; Type B, 0.33–0.67 inclusive; and Type C, <0.33. These boundaries are provisional and would need to be derived and validated in clinical trials. **(A)** Type A, lymphatic-predominant. Tumor emboli occupy multiple PD-L1-expressing lymphatic vessels, with little invasion of blood vessels. Most invaded vessels are lymphatic, and the incremental contribution expected from the anti-VEGF-A arm is hypothesized to be smaller. **(B)** Type B, balanced. Both lymphatic and blood vessels are invaded in comparable proportions. The benefit of the bispecific antibody over either single agent is predicted to be greatest in this group. **(C)** Type C, vascular-predominant. Tumor cells occupy D2-40-negative, CD31-positive blood vessels within a VEGF-A-rich vascular microenvironment, with little lymphatic invasion. Most invaded vessels are blood vessels, and the incremental contribution expected from the anti-PD-L1 arm is hypothesized to be smaller. Created in BioRender. Kern K. (2026).

As shown in **Figure 1**, the LVR classifies LVI-positive tumors as lymphatic-predominant (Type A), balanced (Type B), or vascular-predominant (Type C) using D2–40 and CD31 immunohistochemistry (32, 33). These groups are hypothesized to differ in the benefit derived from a PD-L1×VEGF-A bispecific antibody relative to the corresponding single-agent therapies. Type B tumors are predicted to derive the greatest benefit because invasion of both vessel types places tumor cells within the spatial environments relevant to both antibody arms: PD-L1-expressing lymphatic endothelium (27) and the VEGF-A-rich vascular microenvironment (13).

In the spatial model presented, each target of the antibody’s functional arms occupies a defined location: PD-L1 on the lymphatic endothelium (27) and VEGF-A in the perivascular niche (13). The LVR is the ratio of invaded lymphatic vessels to invaded blood vessels, L:V; for analysis and classification, it is expressed as the lymphatic fraction L/(L+V), where L is the number of invaded lymphatic vessels and V is the number of invaded blood vessels. The proposed initial thresholds are Type A, >0.67; Type B, 0.33–0.67 inclusive; and Type C, <0.33. These thresholds are provisional; the values that best separate responders from nonresponders would need to be derived in retrospective studies and prespecified for prospective validation, using the counting protocol and rules for ambiguous vessels described below.

The prediction depends on which vessel type the invading tumor cells occupy. In lymphatic-predominant tumors (Type A, **Figure 1A**), tumor emboli occupy mostly D2-40-positive vessels lined by PD-L1-expressing lymphatic endothelium (27), such that the incremental benefit expected from VEGF-A blockade is diminished. In vascular-predominant tumors (Type C, **Figure 1C**), tumor cells breach D2-40-negative, CD31-positive vessels within a VEGF-A-rich perivascular niche (13), where VEGF-A induces endothelial FasL expression that selectively kills infiltrating CD8 T cells (14). In this setting, the incremental contribution expected from PD-L1 checkpoint blockade at the lymphatic endothelial surface is reduced. In contrast, in a tumor with a balanced LVR (Type B, **Figure 1B**), invading tumor cells occupy both vessel types in comparable numbers, so that both biological programs contribute to malignant progression. Under this hypothesis, blocking both with a single molecule is predicted to produce the greatest anti-tumor benefit compared to either single agent alone.

## Applicability to PD-L1-directed bispecific antibodies

The spatial model in **Figure 1** applies specifically to bispecific antibodies with a PD-L1×VEGF-A format. VEGF-A concentrates in the perivascular niche (13), whereas PD-L1 is expressed on tumor-associated lymphatic endothelium (27); the vascular and lymphatic components of LVI therefore spatially correspond to the two targets of these antibodies. Pumitamig (BNT327/PM8002), the clinical exemplar, is in phase 2 trials in small cell lung cancer and triple-negative breast cancer (26). PD-1, by contrast, is expressed on T cells and other immune populations that traffic throughout the tumor (28). Because PD-1 has no comparably fixed spatial relationship to either vessel type, the model does not extend to PD-1×VEGF-A agents such as ivonescimab.

## Lymphangiogenesis, vascular remodeling, and immune regulation

The lymphatic and blood vasculatures are governed by separate ligand programs: VEGF-C and VEGF-D act through VEGFR-3 on lymphatic endothelium, whereas VEGF-A acts on blood vessels (37). Primary tumors secrete VEGF-C and VEGF-D, which drive lymphangiogenesis and expand the peritumoral lymphatic vasculature (35–37). In co-culture, Acevedo-Acevedo et al. showed that breast cancer cell lines activate lymphatic endothelial cells (LECs) to upregulate lymphangiogenesis-associated and inflammatory genes, including VEGFC, CXCL8, and NFKB1 (38). Comparable interactions appear in three-dimensional and microfluidic models incorporating interstitial flow and VEGF-A/C signaling, in which LECs contribute to tumor progression (39–42), and VEGF-C and VEGF-D levels correlate with nodal involvement and reduced survival in breast and other solid tumors (35–37, 43). LVI therefore samples a VEGF-remodeled microenvironment whose lymphatic and blood vessels arise from separate ligand programs and present the two targets of a PD-L1×VEGF-A bispecific antibody at different sites.

Abnormal vasculature produced by VEGF signaling shapes immune function within the tumor. Disordered tumor vessels restrict effector T-cell infiltration, promote retention of immunosuppressive myeloid cells, and alter the distribution of antigen and checkpoint molecules, each of which influences responsiveness to checkpoint blockade (13–16). Liu et al. reviewed evidence that vascular normalization reprograms the perivascular environment to favor T-cell infiltration and effector function (44). Vascular invasion, scored as tumor within a D2-40-negative, CD31-positive vessel, identifies tumor entry into the blood circulation within a microenvironment characterized by VEGF-A signaling and the disordered vessel architecture that drives immune exclusion (13–16). The anti-VEGF arm of the bispecific antibody acts on VEGF-A within this vascular microenvironment.

Lymphatic endothelial PD-L1 provides the spatial basis for the lymphatic component of the hypothesis. Tumor-associated lymphatic endothelial cells upregulate PD-L1, suppressing T-cell activation at the lymphatic-tumor interface (27), while interferon-γ released by effector T cells further increases immunosuppressive marker expression on these cells (45). A tumor cell within a lymphatic vessel is therefore in direct contact with PD-L1-expressing lymphatic endothelium (27). Standard clinical PD-L1 assays, including 22C3 and SP263, score PD-L1 on tumor and immune cells but not on lymphatic endothelium (29); quantification of lymphatic endothelial PD-L1 would require dual D2–40 and PD-L1 staining. In human melanoma, immune parameters vary with the expression of lymphatic markers, linking lymphatic content to an immunosuppressive microenvironment (46).

Clinical experience with combination regimens using distinct PD-(L)1- and VEGF-directed agents provides the therapeutic basis for engaging both pathways in a single molecule (17). In IMbrave150, atezolizumab combined with bevacizumab reduced the risk of death compared with sorafenib in unresectable hepatocellular carcinoma (hazard ratio, 0.58), with 12-month overall survival of 67.2% versus 54.6% (19); in the updated analysis, median overall survival was 19.2 versus 13.4 months (47). In IMpower150, atezolizumab combined with bevacizumab and chemotherapy improved median overall survival to 19.2 months versus 14.7 months with bevacizumab plus chemotherapy (hazard ratio, 0.78) (20). In KEYNOTE-426, pembrolizumab combined with the VEGFR inhibitor axitinib improved overall survival (hazard ratio, 0.53), progression-free survival (15.1 versus 11.1 months), and objective response rate (59% versus 36%) compared with sunitinib (21). These studies support the clinical benefit of combined PD-(L)1 and VEGF pathway inhibition (18) and provide the basis for bispecific antibodies that engage both pathways in a single molecule. Patient selection for these agents nevertheless remains based on PD-L1 scoring in tumor and immune cells, without measurement of lymphatic endothelial PD-L1 or a vascular variable corresponding to the VEGF-A-directed arm.

## Why lymphatic and vascular invasion should be reported separately

The principal limitation of routine LVI reporting is that lymphatic invasion (LI) and vascular invasion (VI) are combined into a single present-or-absent designation, although they represent distinct routes of dissemination, occur at different frequencies, and may have different implications for treatment response (32, 33). LI denotes tumor entry into lymphatic vessels draining to regional nodes, whereas VI denotes tumor entry into blood vessels and the systemic circulation. VI is identified as tumor within a D2-40-negative, CD31-positive vessel and is distinct from microvessel density, which measures angiogenesis rather than invasion (48). A binary LVI designation therefore obscures the relative contributions of LI and VI and any relationship between that balance and response to immunotherapy, anti-angiogenic therapy, or their combination.

Reliable separation of LI from VI cannot always be achieved on hematoxylin and eosin (H&E) staining alone. Thin-walled lymphatic vessels and small venules may be morphologically similar (1), while retraction artifact around tumor nests can mimic true invasion (2). In a colorectal series in which pathologists applied their own criteria, interobserver agreement was only fair for small-vessel invasion (κ = 0.28) and lower for large-vessel invasion (κ = 0.18); adding D2–40 and CD31 immunohistochemistry without a shared scoring protocol did not improve agreement (49). In early rectal cancer, diagnostic variability altered the recommended treatment in 11% of patients (50). These findings indicate that separating LI from VI requires both endothelial markers and defined criteria for their interpretation.

Immunohistochemistry can distinguish the two vessel types and confirm genuine invasion when applied according to explicit scoring rules. D2–40 is the discriminating marker because CD31 labels both lymphatic and blood vascular endothelium (32, 33). A D2-40-positive vessel is classified as lymphatic, whereas a D2-40-negative, CD31-positive vessel is classified as a blood vessel. PROX1 and LYVE1 can serve as complementary lymphatic markers when D2–40 staining is equivocal. Because no single marker identifies lymphatic endothelium reliably across all tissues and conditions, a marker panel is recommended (53). Podoplanin, the antigen recognized by D2-40, also stains mesothelium, cancer-associated fibroblasts, and some tumor cells (53, 54); dual or multiplex immunostaining can resolve vessel identity when the single-marker pattern is ambiguous and improve reproducibility across observers (34). Immunohistochemistry also detects LVI more consistently than H&E alone (33). With defined criteria, it can reproducibly distinguish lymphatic from blood vessels (32, 34), whereas the absence of a shared scoring protocol accounts for the failure of immunohistochemistry to improve agreement in the study by Harris et al. (49).

Reliable vessel classification does not, however, determine how the extent or distribution of invasion should be reported. Early attempts to grade LVI according to the number and distribution of involved vessels were not standardized, and routine assessment still lacks validated grading criteria (51, 52). Current reporting therefore captures neither the extent of invasion nor the balance between its lymphatic and vascular components. No standardized method for scoring the LVR yet exists; the next section proposes such a method, whose reproducibility would require validation before the LVR could enter clinical practice.

Once the two vessel types are identified separately, the remaining question is whether invasion carries information beyond the abundance of the vessels themselves. Lymphatic endothelial PD-L1 and perivascular VEGF-A are present even in tumors without LVI (13, 27), so the substrates for both antibody arms do not depend on invasion. Vessel density and vessel invasion nevertheless measure different properties. Density measures the vascular routes available to the tumor, whereas invasion identifies the routes the tumor has entered, requiring migration, matrix degradation, endothelial contact, and survival within the lumen. In node-negative, chemotherapy-naive breast cancer, lymphatic vessel density and LVI remained independent prognostic factors in the same multivariate model, indicating that invasion is not a surrogate for vessel number (55). Evidence that an entered vessel can carry information beyond vessel abundance also comes from the tumor microenvironment of metastasis (TMEM), a contact-defined structure comprising a tumor cell, a macrophage, and an endothelial cell. TMEM predicts distant recurrence in estrogen-receptor-positive, HER2-negative breast cancer independently of classical clinicopathologic variables (56). To the author’s knowledge, no equivalent contact-defined structure has been described for lymphatic vessels. These findings support evaluating invasion and vessel abundance as related but noninterchangeable measurements.

The distinction can be tested using the same immunostained section. Because D2–40 and CD31 identify vessels whether invaded or not, four counts can be obtained: total lymphatic vessels, total blood vessels, invaded lymphatic vessels, and invaded blood vessels. These counts yield the LVR; the lymphatic fraction of all vessels, calculated as the number of lymphatic vessels divided by the total number of lymphatic and blood vessels; and the proportion of each vessel type invaded by tumor. Direct comparison of these measures will determine whether the LVR carries predictive information beyond vessel abundance, which is the first question addressed by the validation plan below.

## A framework for scoring component-directed LVI

The proposed framework defines the LVR as the ratio of invaded lymphatic vessels to invaded blood vessels, L:V, and expresses it for analysis as the lymphatic fraction L/(L+V) within an LVI-positive tumor. When LVI is identified or suspected on routine H&E, D2–40 and CD31 immunohistochemistry are used to confirm intravascular tumor and classify each involved vessel as lymphatic (D2-40-positive) or blood vascular (D2-40-negative, CD31-positive) (32, 33). Equivocal D2–40 staining is adjudicated with PROX1, a transcription factor expressed in lymphatic endothelium, or LYVE1, a lymphatic endothelial surface receptor (53). Vessels that remain unclassifiable are recorded as indeterminate and excluded. Where additional specificity is required, VEGFR3 can support lymphatic identity, ERG can confirm endothelial origin, and CD34 can serve as an alternative blood-vessel marker. Because no single marker identifies lymphatic endothelium reliably across all tissues, vessel assignment is based on a panel rather than on any marker alone (53).

After vessel identity is established, invaded lymphatic and blood vessels are counted within the hotspot containing the greatest number of LVI foci. Prespecifying this field standardizes the area assessed across observers and follows the microvessel density convention of counting within the field of greatest vascular density (48). The same field is also used to count all lymphatic and blood vessels, whether invaded or not. These four counts permit comparison of the LVR with the lymphatic fraction of all vessels and with the proportion of each vessel type invaded by tumor. The LVR is analyzed as a continuous variable and provisionally classified as Type A (lymphatic-predominant), Type B (balanced), or Type C (vascular-predominant). Because validated category boundaries do not yet exist, the thresholds shown in **Figure 1** would be derived in a training cohort and prespecified before testing in an independent validation cohort, thereby limiting bias in evaluation of the LVR.

The LVR is proposed to predict the magnitude of benefit from a PD-L1×VEGF-A bispecific antibody relative to the component single agents. The predicted treatment effect and its mechanistic basis for each LVR category are described in the LVI Component Hypothesis section above.

Any predictive analysis must also account for the established prognostic significance of vascular invasion. Extensive vascular invasion is associated with hematogenous dissemination and reduced survival across solid tumors (3–5); an extreme LVR may therefore identify aggressive tumor biology, sensitivity to treatment, or both. Prospective validation is required to distinguish these possibilities. Because the ratio cannot be calculated in the absence of invaded vessels, LVI positivity remains a prerequisite for stratification by the LVR.

## Molecular and immune features of LVI-positive tumors relevant to immunotherapy

Beyond its established prognostic association, LVI has been linked to molecular features that may influence response to checkpoint blockade. In The Cancer Genome Atlas, LVI-positive breast tumors had greater genome copy-number aberration, aneuploidy, homologous recombination defects, and a strong cell-proliferation signature, although LVI was not accompanied by increased immune infiltration or cytolytic activity in that cohort (30). CCNB1 (cyclin B1) overexpression is associated with high tumor grade, hormone-receptor negativity, and HER2 positivity in LVI-positive breast cancer (57). Tumor mutational burden correlates with response rate and survival after PD-1/PD-L1 blockade across solid tumors (58, 59), and LVI-positive non-small cell lung cancers have higher mutational burden than LVI-negative tumors (31).

Other molecular programs associated with LVI affect PD-L1 expression and T-cell access to the tumor. ISG15, a type I interferon-stimulated modifier, is overexpressed in LVI-positive, high-grade breast cancers and is associated with CD8, FOXP3, and CD68 immune-cell markers and with shorter survival (60). Sustained interferon signaling can also upregulate PD-L1 as a mechanism of adaptive resistance to checkpoint blockade. In pancreatic ductal adenocarcinoma, LVI-positive tumors are enriched for TGF-β signaling, which promotes stromal fibrosis and restricts T-cell infiltration (61), while LVI-positive breast cancers constitute a distinct transcriptomic subtype with independent prognostic value (62).

Evidence from non-small cell lung cancer links LVI more directly to biomarkers used in immunotherapy selection. In a prospectively profiled resection cohort, LVI was associated with higher tumor mutational burden and, on multivariable analysis, with concurrent elevation of tumor mutational burden and PD-L1 expression (odds ratio, 3.53) (31). Tumors with high mutational burden and PD-L1 expression also had greater intratumoral densities of CD3-positive and CD8-positive T cells and PD-L1-positive macrophages. The cohort included only 55 resected tumors, and the association requires confirmation.

These findings suggest that LVI-positive tumors may contain both features favoring response to checkpoint blockade and mechanisms that restrict that response. Mutational burden, PD-L1 expression, and T-cell infiltration provide the immunologic substrate, whereas TGF-β signaling, disordered vasculature, and stromal remodeling impede access to it. This combination provides a rationale for adding VEGF-A inhibition, because vascular normalization may improve T-cell infiltration in tumors that already contain antigen and effector T cells (13, 44).

The proposed value of the LVR therefore depends on whether it contributes information beyond PD-L1 expression and tumor mutational burden. These biomarkers characterize the immune axis, whereas no biomarker in routine clinical use characterizes the angiogenic axis engaged by the VEGF-A-directed arm. Whether the LVR is independent of the PD-L1 tumor proportion score and tumor mutational burden is a prespecified question in the validation plan below.

The biological content of an invaded vessel may also carry information beyond the presence of LVI. In colorectal cancer with LVI, high expression of the adhesion molecule CDH17 within tumor emboli independently predicts mortality, approximately doubling the risk of death compared with low expression (63). This finding indicates that molecular features of the intravascular tumor compartment may refine the prognostic information conveyed by invasion.

The route of tumor dissemination may likewise be associated with response to checkpoint blockade. Among 92 patients with gastric cancer treated with nivolumab, those whose disease spread predominantly through lymphatic and nodal routes had longer median overall survival than those with hematogenous or peritoneal spread (35.1 months versus 4.66–5.80 months) and higher objective response rates (64). The comparison is confounded because hematogenous and peritoneal dissemination may indicate more advanced disease and a poorer underlying prognosis. Nevertheless, the finding is consistent with the possibility that tumors spreading through lymphatic vessels, whose endothelium expresses PD-L1 (27), differ immunologically from tumors spreading through the bloodstream.

## Toward prospective evaluation of LVI in clinical trials

Validation has two components: determining whether the LVR is distinct from vessel abundance and established immunotherapy biomarkers, and testing whether it modifies treatment benefit from PD-L1×VEGF-A blockade. Neither analysis has yet been reported.

D2–40 and CD31 staining provide four counts from the same tissue section: total lymphatic vessels, total blood vessels, invaded lymphatic vessels, and invaded blood vessels. These counts yield the LVR, the lymphatic fraction of all vessels, and the proportion of each vessel type invaded by tumor. The abundance-based measures are necessary comparators because they test whether the distribution of invaded vessels merely reflects the relative availability of lymphatic and blood vessels or whether preferential invasion of one compartment contributes additional information. PD-L1 and VEGF-A expression would also be scored independently of invasion. Because LVI has been associated with elevated PD-L1 expression and tumor mutational burden, the LVR must also be evaluated for independence from the PD-L1 tumor proportion score and tumor mutational burden (31). Together, these analyses would determine whether the LVR is analytically distinct from vessel abundance, pathway expression, and biomarkers already used in immunotherapy selection.

Clinical validation requires outcome-linked archival tissue from trials of PD-L1 and VEGF-A blockade. Trials of pumitamig (BNT327/PM8002) provide the setting in which to test the hypothesis in the bispecific format for which it was developed (26). Completed trials of atezolizumab combined with bevacizumab can provide preliminary evidence for the underlying principle that the LVR modifies benefit from concurrent PD-L1 and VEGF-A inhibition. IMbrave150 in hepatocellular carcinoma (19) and IMpower150 in non-small cell lung cancer (20) included randomized comparator arms and therefore permit a treatment-by-LVR interaction analysis. The ATRACTIB trial in triple-negative breast cancer was single-arm and enrolled a predominantly PD-L1-negative population (22). It can characterize the distribution of LVR values, examine their association with response and survival, and provide effect-size estimates for the design of a subsequent randomized study, although its single-arm design cannot distinguish a predictive association from a prognostic one. The atezolizumab-bevacizumab trials test the relationship between the LVR and combined PD-L1 and VEGF-A blockade delivered as two antibodies; trials of pumitamig test whether the same relationship applies when both activities are incorporated into one molecule. Ivonescimab binds PD-1 and is excluded from this evaluation.

The sequence of retrospective studies would depend on the maturity of the clinical data and the availability of archival tissue. Because the required counts can be obtained from routine pathology material using widely available immunohistochemical stains, these analyses would require neither additional patient procedures nor specialized molecular testing. Once the scoring protocol is standardized, automated digital image analysis could improve reproducibility and support larger retrospective studies.

Definitive validation of the LVR as a predictive biomarker requires a randomized trial with a comparator arm, because an association between the LVR and outcome in a single treatment group may reflect prognosis rather than differential treatment benefit (6). The analysis should include LVI-negative tumors and the three LVI-positive categories, Type A, Type B, and Type C, with LVI status determined from biopsies obtained within a pre-specified interval. Inclusion of the LVI-negative group permits the prognostic effect of LVI presence to be distinguished from any predictive effect of the LVR within LVI-positive tumors. Randomization would compare a PD-L1×VEGF-A bispecific antibody with a monotherapy control, such as anti-PD-L1 monotherapy, consistent with the predictive-marker validation design described by Sargent et al. (7). The primary analysis would test a pre-specified treatment-by-LVR interaction, with the LVR analyzed as a continuous variable to preserve statistical power and the three categories used for stratification and interpretation. Objective response rate, progression-free survival, and overall survival would serve as clinical outcomes.

The pattern of treatment effects would determine the interpretation of the LVR. A similar benefit from the bispecific across the full range of LVR values would argue against a predictive effect. Differences in outcome by LVR that occur similarly in both treatment arms would support prognostic, rather than predictive, value. Absence of both a treatment interaction and an independent association with outcome would indicate that the LVR is uninformative (6). Because interaction tests generally have less statistical power than main-effect analyses, a non-significant interaction would be interpretable only in an adequately powered study. Any positive predictive finding would require replication in an independent trial before the LVR could be considered clinically validated (8).

## Discussion

The LVI Component Hypothesis proposes that the relative distribution of tumor invasion between lymphatic and blood vessels may identify patients most likely to benefit from a PD-L1×VEGF-A bispecific antibody. A balanced LVR indicates invasion of both vessel types and is hypothesized to identify tumors in which both antibody arms contribute substantially to treatment effect. As either lymphatic or vascular invasion becomes predominant, the incremental benefit contributed by the arm directed against the less represented component is hypothesized to decline.

The LVR is intended to provide an angiogenic measure that is absent from current patient-selection strategies for this class. Its assessment builds on the presence of LVI reported in routine pathology and uses D2–40 and CD31, both routinely used immunohistochemical markers. Component-directed LVI can therefore be evaluated in archived trial tissue using histopathology and immunohistochemistry, without prospective specimen collection or specialized molecular assays.

If validated, the LVR could have applications in both trial enrichment and treatment selection. In clinical trials, enrichment for balanced tumors could increase the contrast between the bispecific antibody and its component single agents by concentrating enrollment in the group hypothesized to derive the greatest incremental benefit from dual blockade. In clinical practice, the LVR might inform de-escalation when the anticipated contribution of one antibody arm is small relative to its toxicity. A patient with a lymphatic-predominant tumor might derive limited additional benefit from VEGF-A blockade and could potentially receive anti-PD-L1 monotherapy, avoiding hypertension, proteinuria, hemorrhage, and thromboembolism associated with VEGF-A inhibition (18). Conversely, a patient with a vascular-predominant tumor might derive limited benefit from the checkpoint arm and could potentially avoid immune-related adverse events (18). Either application would require prospective evidence that omission of one arm does not materially reduce treatment benefit.

Existing oncology trial materials provide an initial setting in which to evaluate the hypothesis. Archived tissue from trials of PD-L1×VEGF-A bispecific antibodies and from completed trials of atezolizumab with bevacizumab could undergo retrospective component-directed LVI assessment. The bispecific trials would evaluate the hypothesis in the single-molecule format for which it was developed, whereas the atezolizumab-bevacizumab trials would provide evidence concerning combined inhibition of the same two pathways with separate antibodies. Variation in fixation and block age would require central pathology review and uniform application of the scoring panel. Although pretreatment tissue is generally collected in clinical trials, the number of LVI-positive cases within an individual study may be insufficient for a powered biomarker analysis. A retrospective association could nevertheless provide estimates of LVR prevalence and effect size for the design of a subsequent randomized prospective trial.

## Limitations of the LVI component hypothesis

The principal limitations concern the biological model, the absence of predictive clinical data, and the reproducibility of component-directed LVI assessment.

The lymphatic component of the model remains incompletely characterized. Lymphatic vessels also provide routes of T-cell egress, and tumor-reactive T cells leave tumors through lymphatics in a process regulated by antigen encounter (65). The net balance between T-cell retention and egress in LVI-positive tumors is unknown. It is also uncertain whether D2-40-positive lymphatic endothelium consistently expresses PD-L1 and whether lymphatic endothelial PD-L1 provides information independent of the tumor-cell and immune-cell PD-L1 scores used clinically. Dual D2–40 and PD-L1 staining would allow both questions to be examined directly.

The proposed relationship between the LVR and treatment benefit also depends on the contribution of each antibody arm being influenced by the relative involvement of its corresponding vascular compartment. PD-L1 and VEGF-A remain present in tumors across the range of LVR values, and the activity of an antibody arm may extend beyond the immediate site of target expression through avidity, altered pharmacokinetics, or systemic immune effects. Substantial contributions from these mechanisms could produce similar responses in balanced and markedly unbalanced tumors, contrary to the predicted LVR-treatment relationship.

Clinical evidence for a predictive association is currently indirect. The reported association of LVI with tumor mutational burden and PD-L1 expression arises largely from non-small cell lung cancer (31). Evidence that vessel invasion and vessel density carry different information comes from prognostic cohorts rather than patients treated with PD-L1×VEGF-A bispecific antibodies, and its relevance to differential treatment benefit remains unknown. LVI-negative tumors may still contain PD-L1-expressing lymphatic vessels and blood vessels within VEGF-rich niches; a density-based ratio could therefore perform as well as, or better than, an invasion-based measure in some settings.

The LVR itself does not account for the number of vessels available for invasion. Tumors with the same LVR may differ substantially in the proportion of each vascular compartment invaded, depending on the underlying abundance of lymphatic and blood vessels. The four counts specified above, total and invaded lymphatic and blood vessels, permit concurrent calculation of the LVR, the lymphatic fraction of all vessels, and the proportion of each vessel type invaded. Comparing these measures in the same trial population will determine whether the pattern of invasion predicts differential treatment benefit beyond vessel abundance alone.

Reproducible classification of LI and VI presents an additional methodological constraint. Interobserver agreement for LVI assessment on standard H&E is poor (49), reflecting both the morphological similarity of thin-walled lymphatic vessels and small venules and the difficulty of distinguishing true invasion from fixation-related retraction artifact (2). Evaluation of the hypothesis will therefore require defined scoring criteria, D2–40 and CD31 immunohistochemistry, a confirmatory marker panel where vessel identity remains equivocal, and validation of interobserver reproducibility (53).

The LVR has no validated threshold, has not been used for treatment selection, and has not been tested for interaction with the benefit of a PD-L1×VEGF-A bispecific antibody. Its clinical relevance will depend on reproducible measurement, demonstration that it provides information beyond vessel abundance and established immunotherapy biomarkers, and confirmation of a treatment-by-LVR interaction in an independent randomized trial. If validated, the LVR could refine treatment selection by identifying patients most likely to benefit from a PD-L1×VEGF-A bispecific antibody and those in whom it is unlikely to improve outcome over the corresponding single-agent therapy.

## Data Availability

The original contributions presented in the study are included in the article/supplementary material, further inquiries can be directed to the corresponding author/s.
